# Cost-effectiveness of hetrombopag, eltrombopag, and avatrombopag for chronic immune thrombocytopenia in China: a cost-utility analysis

**DOI:** 10.3389/fpubh.2026.1763592

**Published:** 2026-02-19

**Authors:** Shanshan Jin, Zhengqiang Hu, Yuancheng Jin, Wang Lv, Zhujin Song, Su Zeng, Miaolian Wu

**Affiliations:** 1 Department of Pharmacy, The Fourth Affiliated Hospital of School of Medicine, International School of Medicine, International Institutes of Medicine, Zhejiang University, Yiwu, China; 2 College of Pharmaceutical Sciences, Zhejiang University, Hangzhou, Zhejiang, China; 3 Research Center for Clinical Pharmacy, Zhejiang University, Hangzhou, Zhejiang, China; 4 Department of Pharmacy, The Children’s Hospital, Zhejiang University School of Medicine, National Clinical Research Center for Child Health, Hangzhou, Zhejiang, China

**Keywords:** avatrombopag, chronic immune thrombocytopenia, cost-utility analysis, eltrombopag, hetrombopag, thrombopoietin receptor agonists

## Abstract

**Objectives:**

Thrombopoietin receptor agonists (TPO-RAs) are standard second-line therapies for chronic immune thrombocytopenia (ITP). Given the introduction of domestic options like hetrombopag, a comparative economic evaluation is essential to inform reimbursement policies in China. This study assessed the cost-effectiveness of hetrombopag, eltrombopag, and avatrombopag for Chinese adults with chronic ITP.

**Methods:**

A hybrid model comprising a decision tree and a Markov process was developed to simulate lifetime costs and health outcomes from the perspective of the Chinese healthcare system. Clinical efficacy parameters were derived from randomized controlled trials and a network meta-analysis (NMA). Utilities and costs were estimated using published literature and local data, respectively. Future costs and quality-adjusted life-years (QALYs) were discounted at 5% annually. The primary outcome was the incremental net monetary benefit (iNMB) at a willingness-to-pay (WTP) threshold of CNY 287,391/QALY. Deterministic and probabilistic sensitivity analyses were performed to evaluate uncertainty.

**Results:**

Over a lifetime horizon, hetrombopag was the lowest-cost strategy (CNY 2,205,717), followed by eltrombopag (CNY 2,214,322) and avatrombopag (CNY 2,379,335). Although avatrombopag yielded the highest QALYs (10.449), hetrombopag (10.335 QALYs) dominated eltrombopag (10.159 QALYs) by providing greater health benefits at lower costs. Compared with avatrombopag, hetrombopag generated a positive iNMB of CNY 140,864, as the substantial cost savings outweighed the marginal reduction in QALYs. Probabilistic sensitivity analysis indicated that hetrombopag had the highest probability of being cost-effective across standard WTP thresholds.

**Conclusion:**

Under current pricing, hetrombopag represents the most cost-effective second-line TPO-RA for adult chronic ITP in China. It dominates eltrombopag and offers a favorable economic profile compared with avatrombopag. These findings support the use of hetrombopag as a preferred option in resource-limited settings.

## 1 Introduction

Chronic immune thrombocytopenia (ITP) is a chronic autoimmune disorder characterized by a low platelet count (<100 × 10^9^/L), leading to an increased risk of bleeding and significantly impaired health-related quality of life (1–4). Beyond the immediate clinical risks of hemorrhage, the burden of ITP includes debilitating fatigue, activity restrictions, and anxiety. These factors impose a substantial economic strain on healthcare systems due to the costs associated with long-term monitoring, hospitalization, and the management of bleeding events (5–9).

The primary goals of ITP management are to increase platelet counts to a safe level, minimize the risk of clinically significant bleeding, and maintain patient quality of life (10). While corticosteroids and intravenous immunoglobulin (IVIg) remain standard first-line therapies, their long-term utility is often restricted by significant adverse effects and high relapse rates upon tapering (10–12). Consequently, a substantial proportion of patients require second-line therapies. In recent years, thrombopoietin receptor agonists (TPO-RAs) have become a standard of care for second-line treatment (11, 13). By stimulating platelet production, TPO-RAs effectively maintain safe platelet counts and reduce bleeding events in patients with chronic ITP (14–16).

Despite their clinical benefits, the high acquisition costs of TPO-RAs present challenges for healthcare resource allocation. While several pharmacoeconomic analyses of TPO-RAs have been conducted (17–20), the available evidence remains fragmented in terms of settings and comparators. Consequently, an economic evaluation that simultaneously compares the three oral TPO-RAs currently relevant to clinical practice in China—including the domestically developed hetrombopag—remains lacking. This uncertainty presents a challenge for policymakers and clinicians in making informed formulary and treatment decisions.

Therefore, the objective of this study was to evaluate the cost-effectiveness of three TPO-RAs—eltrombopag, hetrombopag, and avatrombopag—as second-line treatments for adult patients with chronic ITP from the perspective of the Chinese healthcare system.

## 2 Methods

### 2.1 Model structure

We developed a hybrid economic model consisting of a short-term decision tree and a long-term Markov process to evaluate the cost-utility of three TPO-RAs from the perspective of the Chinese healthcare system. The model simulated a lifetime horizon with a cycle length of 4 weeks. A half-cycle correction was applied to state transitions. Both costs and health outcomes were discounted at an annual rate of 5%, consistent with Chinese pharmacoeconomic guidelines (21).

**Figure 1:**
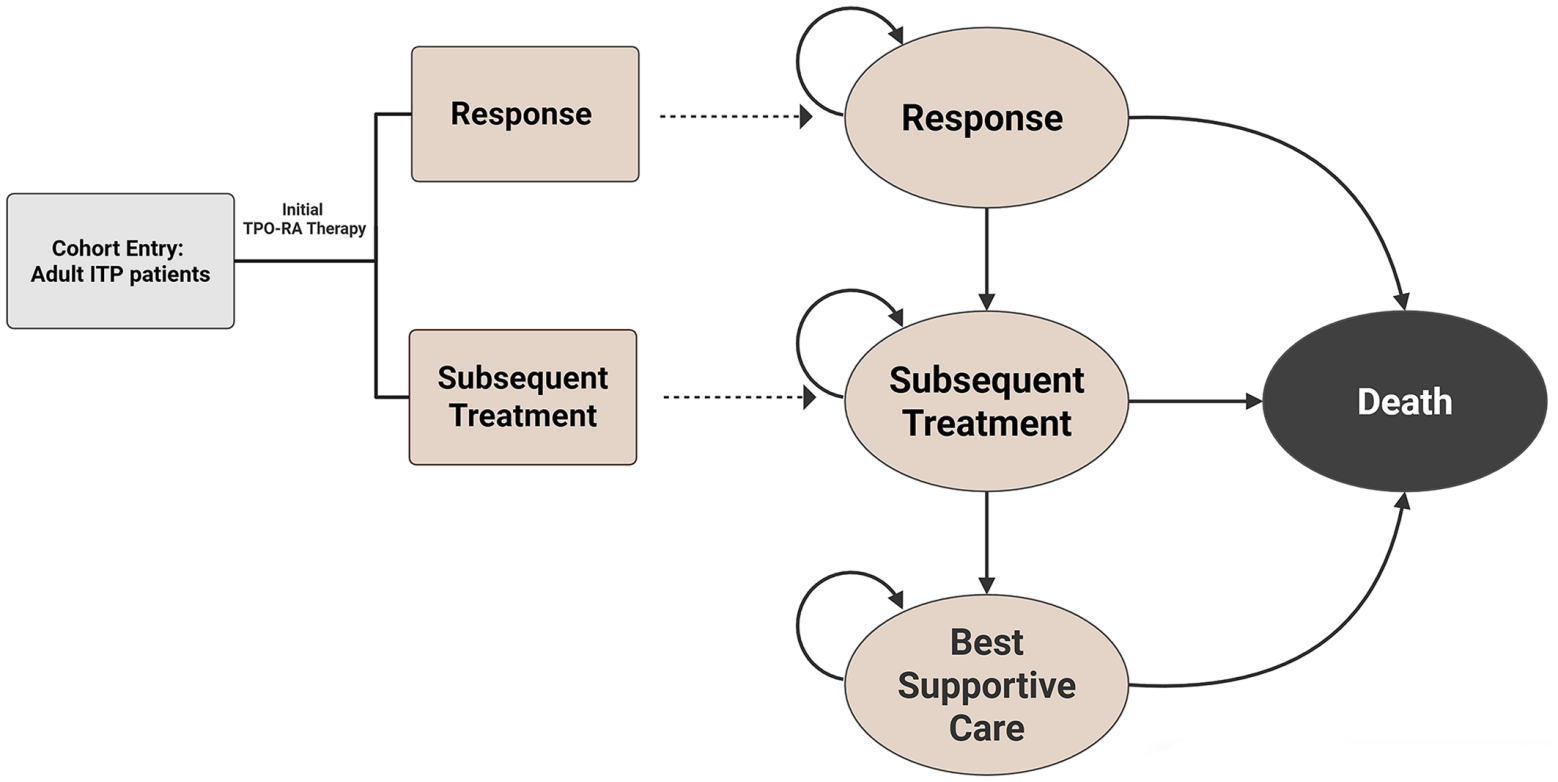
Schematic representation of the model structure. The model comprises a decision tree (left) determining the initial response to TPO-RA therapy and a Markov cohort model (right) simulating long-term health outcomes. The cycle length of the model was 4 weeks. ITP, immune thrombocytopenia; TPO-RA, thrombopoietin receptor agonist.

The model structure comprised two phases. The first phase used a decision tree to simulate the immediate response to initial TPO-RA therapy. Based on the initial outcome, patients entered the second phase, a long-term Markov process with four mutually exclusive health states: “Response,” “Subsequent Treatment,” “Best Supportive Care (BSC),” and “Death.” Patients in the “Response” state (defined as a platelet count >50 × 10^9^/L) continued their current therapy but faced a cyclical risk of loss of response, necessitating a transition to “Subsequent Treatment.” Patients failing subsequent treatment progressed to the “BSC” state. The overall model structure is illustrated in Figure 1.

The four-state structure in the Markov model was chosen to align with the efficacy endpoints available from clinical trials and the platelet-driven treatment pathway standard in Chinese clinical practice. A “remission without treatment” state was excluded because spontaneous remission is rare in this refractory adult population. Similarly, a specific state for discontinuation due to adverse events was not modeled separately, as no statistically significant differences in adverse event rates were observed among the three TPO-RAs (22). Instead, patients discontinuing treatment due to adverse events were captured by the duration of response curves derived from clinical trial data and were assumed to transition to the “Subsequent Treatment” state.

### 2.2 Population and interventions

The model simulated a hypothetical cohort of adult patients with chronic ITP in China who had relapsed or were refractory to first-line therapies (corticosteroids or IVIg). Baseline characteristics were derived from the Phase III clinical trial of eltrombopag in China (23) (Supplementary Table S1).

Three second-line strategies were compared among eltrombopag, hetrombopag, and avatrombopag. To isolate the economic value of the initial TPO-RA therapies, the downstream treatment pathway was standardized. We assumed that all patients failing the initial therapy received a uniform regimen of rituximab combined with recombinant human thrombopoietin (rhTPO).

### 2.3 Clinical inputs

#### 2.3.1 Efficacy parameters

Treatment efficacy was defined by three parameters: initial response rate, time to response, and duration of response. For eltrombopag, the initial response rate was sourced directly from its registrational Phase III trial in China. To model the durability of efficacy, parametric survival models were fitted to patient-level data from the EXTEND long-term extension study. Based on the Akaike Information Criterion (AIC) and Bayesian Information Criterion (BIC), the log-normal distribution was selected as the best-fitting model to extrapolate the long-term duration of response.

Due to the absence of direct head-to-head clinical trials, the relative efficacy of hetrombopag and avatrombopag was derived via a network meta-analysis (NMA) (22). The odds ratios (ORs) from the NMA were applied to the eltrombopag baseline data to estimate the corresponding efficacy parameters for the comparators. Based on published literature (10), the time to response for all three TPO-RAs and subsequent treatments was standardized to one cycle (4 weeks). Similarly, the efficacy of the subsequent treatment (rituximab + rhTPO) was characterized by time to response, initial response rate, and duration of response. The long-term duration was estimated by extrapolating survival functions fitted to digitized Kaplan–Meier curves from the relevant clinical trial (24) (Supplementary Figure S1 and Table S3). Additional parameters are detailed in Table 1.

**Table 1:** Clinical inputs.

Parameter name	Base-case value	Lower value	Upper value	Distribution	Source
Treatment efficacy					
Initial response rate of eltrombopag	57.70%	46.16%	69.24%	Beta	(23)
Mean duration of response, eltrombopag (Cycles)	42.66	34.13	51.19	Lognormal	(16)
OR: Eltrombopag *vs.* Hetrombopag	0.74	0.38	1.43	Lognormal	(22)
OR: Eltrombopag *vs.* Avatrombopag	0.56	0.29	1.09	Lognormal	(22)
Initial response rate of subsequent treatment	79.20%	63.36%	95.04%	Beta	(24)
Mean duration of response of subsequent treatment (cycles)	46.72	37.38	56.07	Lognormal	(24)
Mortality					
SMR for BSC	4.2	1.7	10.0	Lognormal	(26)
Bleeding event probabilities (per cycle)					
Probability of outpatient bleeding (Response / subsequent treatment)	0.0108	0.0086	0.0130	Beta	(20)
Probability of outpatient bleeding (BSC)	0.0381	0.0305	0.0457	Beta	(20)
Probability of inpatient bleeding (BSC)	0.0015	0.0012	0.0018	Beta	(20)

OR, odds ratio; SMR, standardized mortality ratio; BSC, best supportive care.

#### 2.3.2 Bleeding events

Bleeding events were stratified into two categories: minor bleeding (outpatient care) and severe bleeding (hospitalization). Bleeding risk was modeled as a function of the health state. Consistent with prior studies (20), patients in the “Response” and “Subsequent Treatment” states were assumed to have a lower risk and experienced only minor bleeding events. Conversely, patients in the “BSC” state were subject to a higher risk of both minor and severe bleeding events due to uncontrolled platelet counts.

#### 2.3.3 Mortality

Age-specific background mortality rates derived from Chinese national life tables (25) (Supplementary Table S4) were applied to patients in the “Response” and “Subsequent Treatment” states. Patients in the “BSC” state were assigned an elevated mortality risk to reflect the clinical consequences of persistent thrombocytopenia (26).

### 2.4 Costs and resource use

The study considered only direct medical costs, including drug acquisition, routine monitoring, management of bleeding events, and terminal care. Drug dosages were based on mean maintenance doses reported in package inserts and pivotal clinical trials (Supplementary Table S2). Unit costs were derived from local public hospital charge lists or public procurement databases in China and adjusted to 2024 CNY. Detailed cost parameters are presented in Table 2.

**Table 2:** Costs inputs.

Parameter name	Base-case value (CNY)	Lower value (CNY)	Upper value (CNY)	Distribution	Source
Drug acquisition costs (per cycle)					
Eltrombopag	6,365.52	5,092.42	7,638.62	Gamma	Local hospital
Hetrombopag	6,527.92	5,222.34	7,833.50	Gamma	Local hospital
Avatrombopag	11,088.00	8,870.40	13,305.60	Gamma	Local hospital
Rituximab	4,120.00	3,296.00	4,944.00	Gamma	Local hospital
rhTPO	14,026.21	11,220.97	16,831.45	Gamma	Local hospital
Drug administration costs (per cycle)					
TPO-RAs	41.04	32.83	49.25	Gamma	(20)
Rituximab + rhTPO	1,094.40	875.52	1,313.28	Gamma	(20)
Health State Management Costs (per cycle)	463.00	370.40	555.60	Gamma	(20)
Event and terminal care costs					
Outpatient bleeding	132.62	106.10	159.15	Gamma	Local hospital
Intracranial hemorrhage	21,017.80	16,814.24	25,221.36	Gamma	(25)
Gastrointestinal bleeding	8,398.50	6,718.80	10,078.20	Gamma	(25)
Other inpatient bleeding	8,398.50	6,718.80	10,078.20	Gamma	(25)
Terminal care	37,442.00	29,954.00	44,930.00	Gamma	(31)

Due to the lack of data for “other inpatient bleeding”, it was assumed to be equivalent to the cost of gastrointestinal bleeding. TPO-RAs, thrombopoietin receptor agonists; rhTPO, recombinant human thrombopoietin; CNY, Chinese Yuan.

### 2.5 Utilities

Health outcomes were measured in quality-adjusted life-years (QALYs). Given the lack of high-quality utility data specific to the Chinese ITP population, baseline utility values were sourced from a study of UK patients (27). The model assigned baseline utilities according to health states and bleeding events. Detailed utility inputs are provided in Table 3.

**Table 3:** Utility inputs.

Parameter name	Base-case value	Lower value	Upper value	Distribution	Source
Response/subsequent treatment					
No bleeding	0.863	0.457	1.000	Beta	(27)
Minor bleeding	0.734	0.295	0.986	Beta	(27)
BSC					
No bleeding	0.841	0.320	0.999	Beta	(27)
Minor bleeding	0.732	0.294	0.985	Beta	(27)
Intracranial hemorrhage	0.038	0.030	0.046	Beta	(27)
Gastrointestinal bleeding	0.540	0.432	0.648	Beta	(27)
Other severe bleeding	0.540	0.432	0.648	Beta	(27)

BSC, best supportive care.

### 2.6 Economic evaluation and sensitivity analyses

The primary outcome was the incremental net monetary benefit (iNMB), calculated as iNMB = (ΔE × WTP) – ΔC, where ΔE and ΔC represent the incremental effectiveness and costs, respectively. The WTP threshold was set at three times the per capita GDP of China in 2024.

To assess the impact of parameter uncertainty, we performed both deterministic sensitivity analysis (DSA) and probabilistic sensitivity analysis (PSA). In the DSA, key parameters were varied within their confidence intervals or by ±20% of the base-case value, with results presented in tornado diagrams. For the PSA, 5,000 iterations were performed using Monte Carlo simulation. Probability distributions were assigned based on parameter types: beta distributions for probabilities and utilities, gamma distributions for costs, and log-normal distributions for relative effect measures. PSA results were presented using cost-effectiveness acceptability curves and scatterplots.

### 2.7 Key model assumptions

Standardized Subsequent Therapy: Subsequent treatment for all patients failing initial TPO-RA therapy was standardized to a single regimen of rituximab + rhTPO. This approach was adopted to minimize potential bias from heterogeneous downstream pathways.

Extrapolation of response: Long-term duration of response was estimated by extrapolating survival functions fitted to data from clinical trial extension studies.

Average clinical trial dosing: Drug costs were calculated based on the average doses reported in the pivotal clinical trials for each respective agent.

## 3 Results

### 3.1 Base-case analysis

Base-case results over a lifetime horizon are summarized in Table 4. Hetrombopag was the lowest-cost strategy (CNY 2,205,717), followed by eltrombopag (CNY 2,214,322) and avatrombopag (CNY 2,379,335). In terms of effectiveness, avatrombopag yielded the highest health gains (10.449 QALYs), followed by hetrombopag (10.335 QALYs) and eltrombopag (10.159 QALYs).

**Table 4:** Base-case cost-effectiveness results.

Strategy/comparison	Total Cost (CNY)	Total QALYs	Incremental cost (CNY)	Incremental QALYs	iNMB (CNY)
Base results					
Hetrombopag	2,205,717	10.335	—	—	—
Eltrombopag	2,214,322	10.159	—	—	—
Avatrombopag	2,379,335	10.449	—	—	—
Comparisons					
Hetrombopag *vs.* Eltrombopag	—	—	−8,605	0.176	59,275
Hetrombopag *vs.* Avatrombopag	—	—	−173,618	−0.114	140,864
Eltrombopag *vs.* Avatrombopag	—	—	−165,012	−0.290	81,588

CNY, Chinese Yuan; QALYs, quality-adjusted life-years; iNMB, incremental net monetary benefit.

In the comparison between hetrombopag and eltrombopag, hetrombopag was associated with lower total costs (−CNY 8,605) and greater health benefits (+0.176 QALYs). Consequently, hetrombopag dominated eltrombopag, yielding an iNMB of CNY 59,275 at the WTP threshold of CNY 287,391/QALY.

When comparing hetrombopag with avatrombopag, although avatrombopag provided an incremental gain of 0.114 QALYs, it incurred substantial additional costs of CNY 173,618. Conversely, hetrombopag generated significant cost savings that outweighed the QALY difference, resulting in a positive iNMB of CNY 140,864. Similarly, eltrombopag was cost-effective compared with avatrombopag (iNMB: CNY 81,588), as its lower cost profile offset its lower efficacy.

### 3.2 Deterministic sensitivity analysis

The robustness of the base-case results was assessed using DSA. Key drivers are illustrated in tornado diagrams.

**Figure 2:**
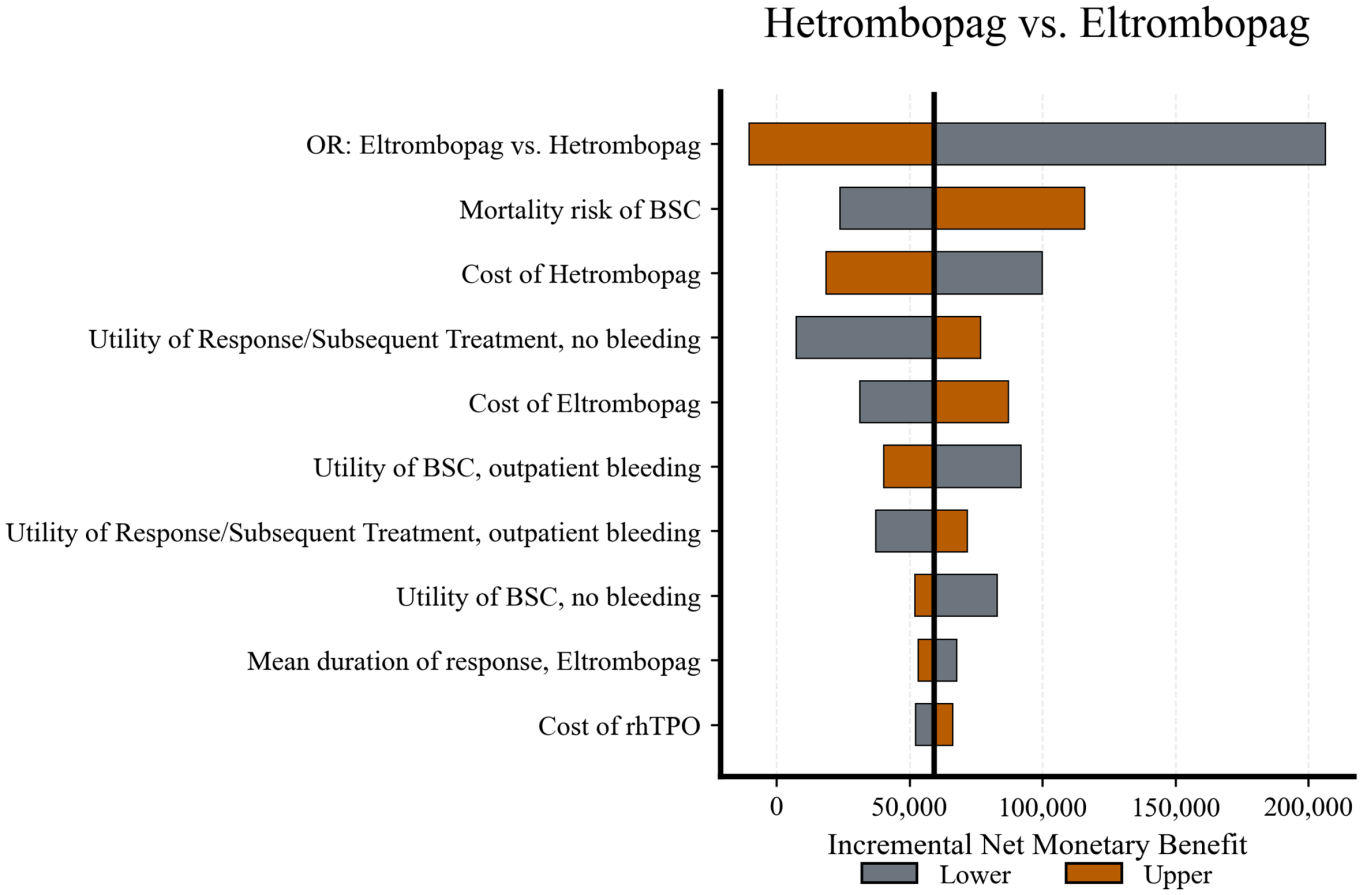
Tornado diagram of deterministic sensitivity analysis: hetrombopag *vs.* eltrombopag. The bar for “OR: Eltrombopag *vs.* Hetrombopag” crosses the zero line, indicating that extreme values for relative efficacy can reverse the base-case conclusion. OR, odds ratio; BSC, best supportive care; rhTPO, recombinant human thrombopoietin.

Hetrombopag *vs.* Eltrombopag (Figure 2): The iNMB was most sensitive to the relative efficacy, specifically the OR of response derived from the NMA. The wide confidence interval for this parameter caused the iNMB to cross zero, indicating that extreme values favoring eltrombopag could reverse the conclusion of dominance. Other parameters, such as BSC mortality risk and drug acquisition costs, influenced the magnitude of the iNMB but did not alter the direction of the results.

**Figure 3:**
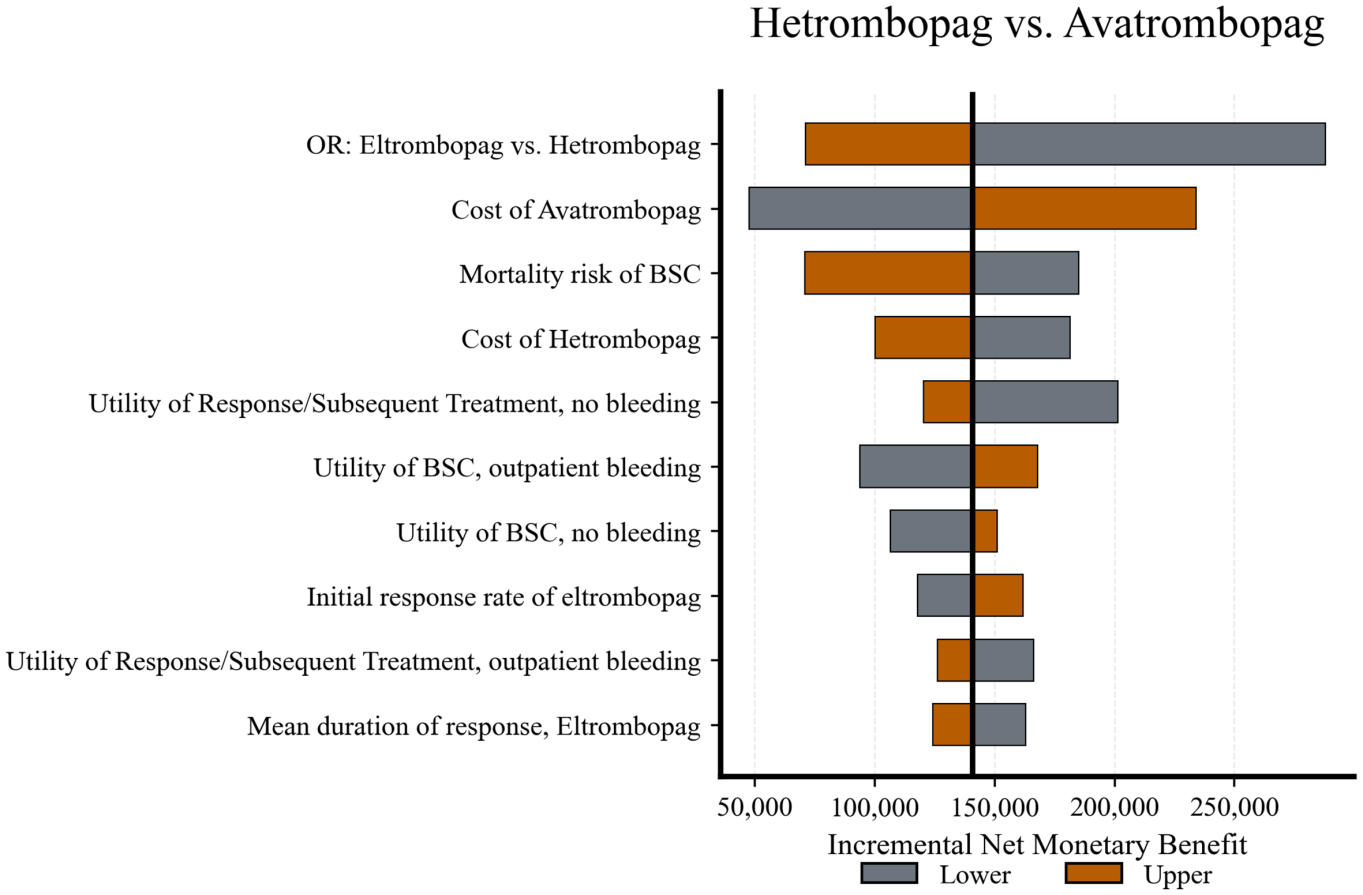
Tornado diagram of deterministic sensitivity analysis: Hetrombopag *vs.* avatrombopag. Variations in the OR and drug acquisition costs have the most significant impact but do not alter the base-case conclusion. OR, odds ratio; BSC, best supportive care.

Hetrombopag *vs.* Avatrombopag (Figure 3): The economic superiority of hetrombopag proved robust. While the OR of response and the acquisition cost of avatrombopag were influential, the iNMB remained consistently positive across the tested ranges. This indicates that hetrombopag remains the cost-effective choice even under conservative assumptions regarding relative efficacy or price reductions for avatrombopag.

**Figure 4:**
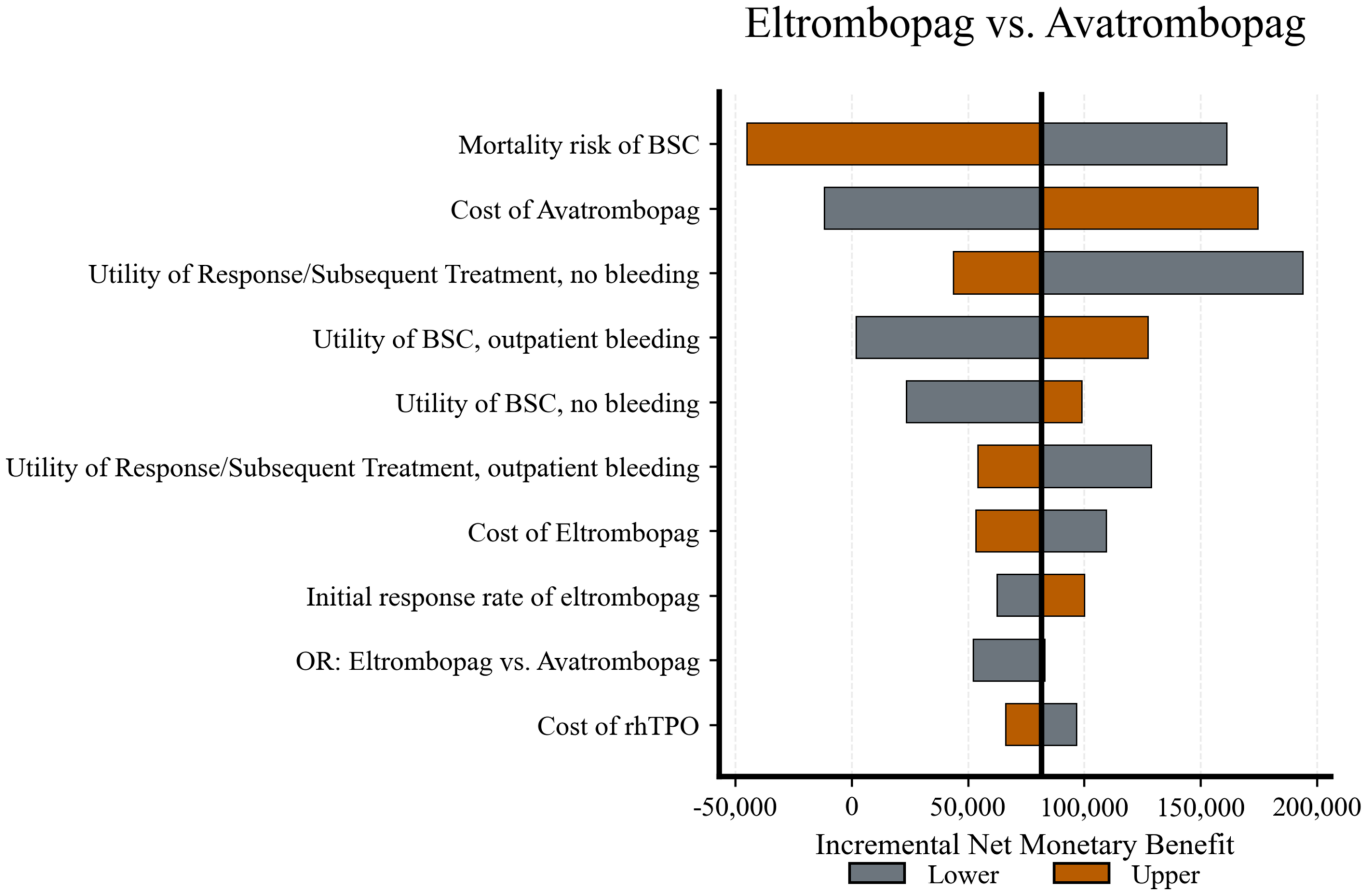
Tornado diagram of deterministic sensitivity analysis: Eltrombopag *vs.* avatrombopag. The iNMB is sensitive to the mortality risk and the cost of avatrombopag; bars for these parameters cross the zero line, indicating potential reversal of the preferred strategy. BSC, best supportive care; OR, odds ratio; rhTPO, recombinant human thrombopoietin.

Eltrombopag *vs.* Avatrombopag (Figure 4): This comparison was sensitive to the BSC mortality risk and the cost of avatrombopag. The iNMB bars for these parameters crossed the zero line, suggesting that specific clinical scenarios or pricing adjustments could render avatrombopag a cost-effective alternative to eltrombopag.

### 3.3 Probabilistic sensitivity analysis

The PSA, based on 5,000 Monte Carlo simulations, corroborated the deterministic findings.

**Figure 5:**
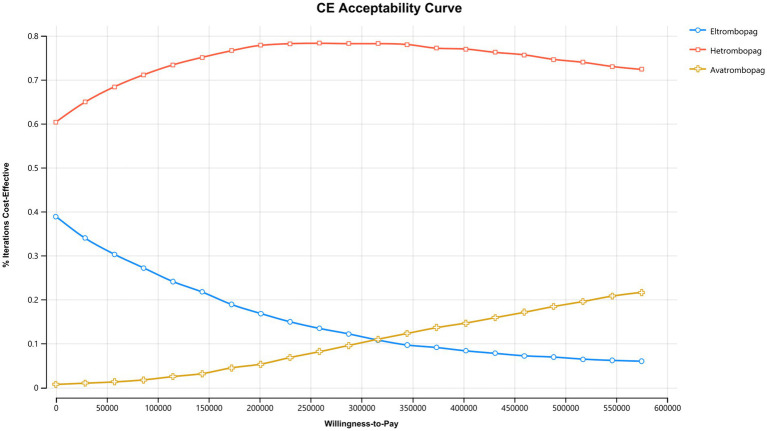
Cost-effectiveness acceptability curve. Hetrombopag remains the strategy with the highest probability of being cost-effective across the standard threshold range.

The Cost-Effectiveness Acceptability Curve (Figure 5) illustrates the probability of each strategy being optimal across a range of WTP thresholds. Hetrombopag consistently maintained the highest probability of cost-effectiveness (approximately 60%–80%). Eltrombopag was the second most likely optimal strategy at lower thresholds, but its probability declined as the WTP increased. Avatrombopag showed a low probability (<25%) of being cost-effective across the standard threshold range.

**Figure 6:**
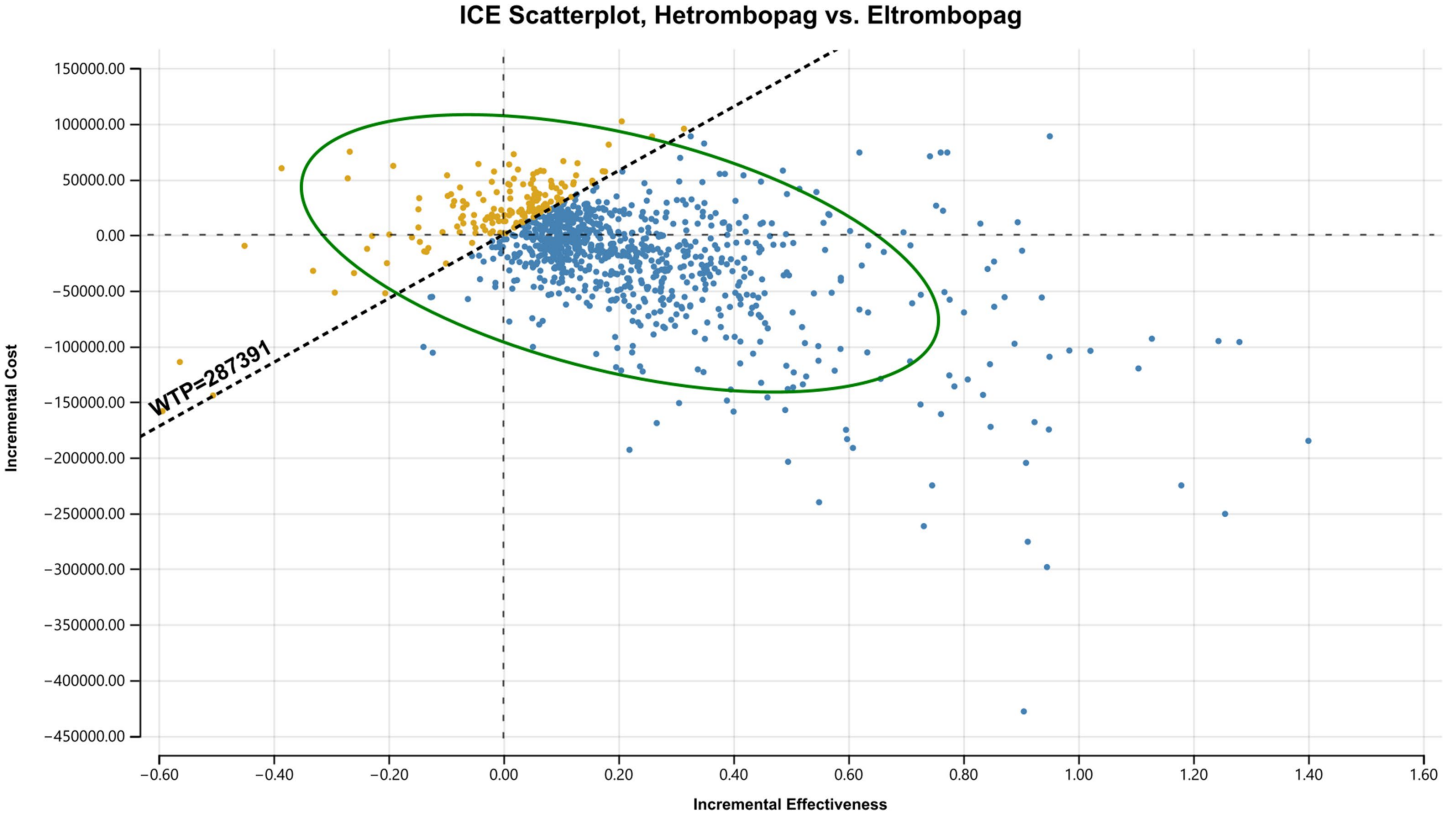
Incremental cost-effectiveness scatterplot: Hetrombopag *vs.* eltrombopag. Each dot represents one Monte Carlo simulation. The dashed line represents the WTP threshold. Points falling below the line indicate that hetrombopag is cost-effective.

**Figure 7:**
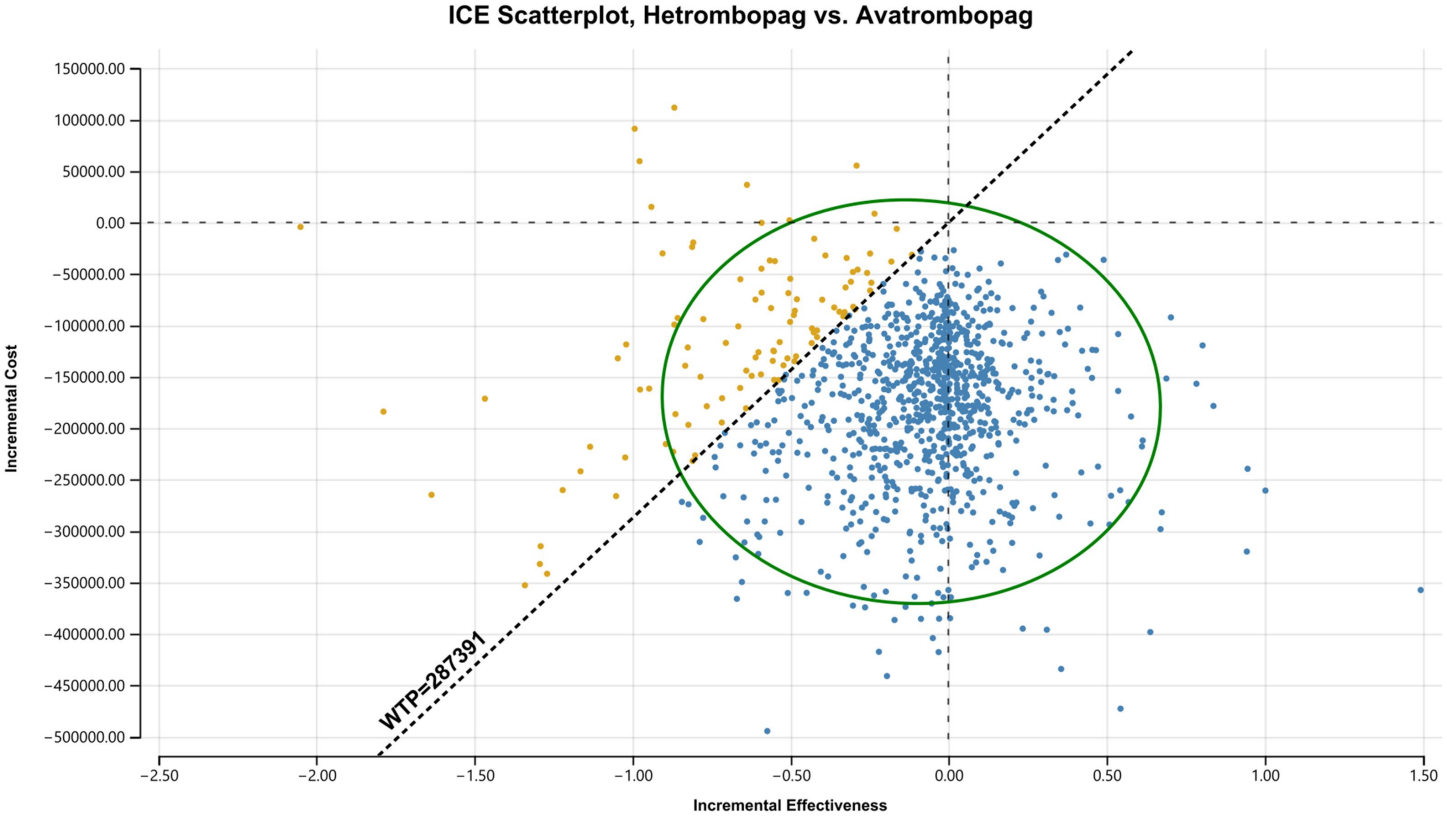
Incremental cost-effectiveness scatterplot: Hetrombopag *vs.* Avatrombopag. Comparison of hetrombopag against avatrombopag. Most points falling below the WTP threshold line indicate that hetrombopag is cost-effective.

**Figure 8:**
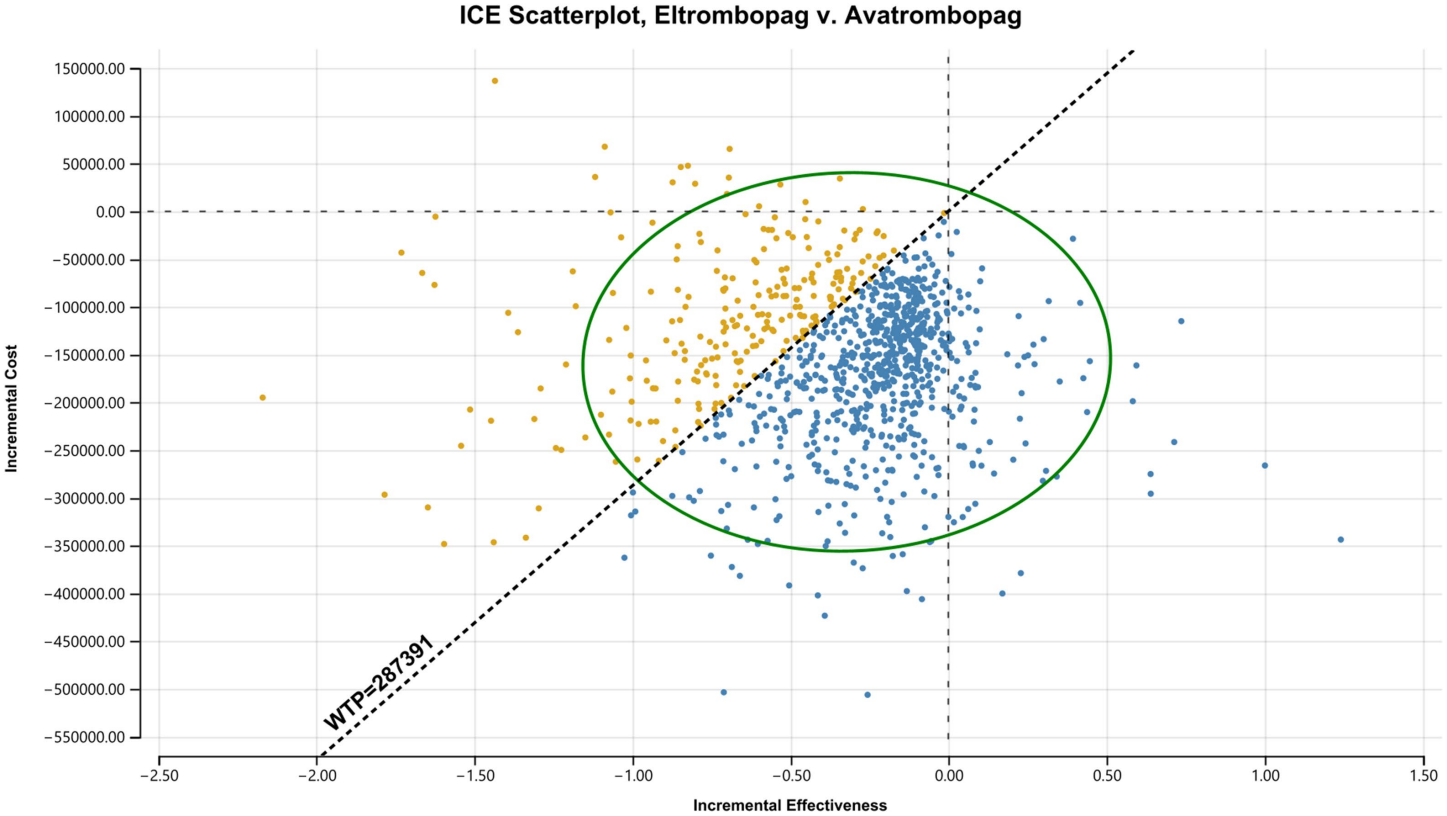
Incremental cost-effectiveness scatterplot: Eltrombopag *vs.* Avatrombopag. Comparison of eltrombopag against avatrombopag. Most points falling below the WTP threshold line indicate that eltrombopag is cost-effective.

Scatterplots (Figures 6–8) further visualized these results. In the comparison between hetrombopag and eltrombopag (Figure 6), the majority of iterations fell within the southeast quadrant (lower cost, higher QALYs), reinforcing the dominance of hetrombopag. Against avatrombopag (Figure 7), simulations clustered in the southwest quadrant (lower cost, lower QALYs) but predominantly fell below the WTP threshold line, confirming that the cost savings of hetrombopag justify the reduction in QALYs under the current Chinese WTP threshold. Finally, the scatterplot comparing eltrombopag and avatrombopag (Figure 8) reinforces the economic advantage of eltrombopag. The majority of iterations clustered in quadrants indicating that eltrombopag represents a cost-effective alternative to avatrombopag.

## 4 Discussion

This study provides the first comparative cost-utility analysis of three oral TPO-RAs—eltrombopag, hetrombopag, and avatrombopag—specifically within the Chinese healthcare system. Our results indicate that hetrombopag is currently the most economically efficient strategy for the second-line treatment of adult ITP. It demonstrated dominance over eltrombopag by providing greater health benefits at a lower cost. Furthermore, compared with avatrombopag, hetrombopag offered a substantial iNMB. Although avatrombopag yielded the highest aggregate QALYs, its incremental cost per QALY significantly exceeded the Chinese WTP threshold, making it economically less favorable under current pricing.

Our findings diverge from international pharmacoeconomic evaluations. A recent UK-based study by Cooper et al. reported that avatrombopag offered superior health outcomes and was cost-effective compared with eltrombopag (18). In contrast, our analysis in China suggests that despite the clinical efficacy advantages of avatrombopag, it is not cost-effective at current local price levels. This disparity underscores the critical role of local acquisition costs in determining value and highlights that conclusions from Western markets cannot be directly extrapolated to the Chinese setting.

To further address economic disparities across Chinese provinces, we evaluated the impact of alternative WTP thresholds. As illustrated in the Cost-Effectiveness Acceptability Curve (Figure 5), hetrombopag maintains the highest probability of being cost-effective across a wide range of WTP values. This is further corroborated by our scenario analysis using a stricter threshold equivalent to the per capita GDP, which confirmed that lowering the threshold solidifies the preference for hetrombopag, as the high incremental cost per QALY of avatrombopag becomes even less acceptable (Supplementary Table S5).

Beyond the specific context of China, this study may offer certain implications for other middle-income countries. First, the hybrid decision tree-Markov model structure developed here could potentially serve as a methodological framework adaptable to other healthcare settings, subject to careful recalibration of local cost and utility inputs. Second, while drug prices differ globally, the economic trade-off discussed in our analysis—weighing the higher acquisition costs of novel agents against the administration burden of lower-cost alternatives—might offer a relevant perspective for policymakers in other resource-limited settings when optimizing their formularies.

Beyond direct acquisition costs, the convenience of administration and its impact on adherence warrant consideration. Our model assumed 100% adherence, which may overestimate real-world effectiveness for regimens with complex dosing requirements. Eltrombopag requires strict dietary restrictions, necessitating administration on an empty stomach (at least 2 h before or 4 h after consuming polyvalent cations). Hetrombopag shares similar but slightly less stringent requirements (administration on an empty stomach with food permitted 2 h post-dose). In contrast, avatrombopag can be taken with food, offering a distinct convenience advantage that may translate to better adherence. In real-world practice, the strict fasting requirements for eltrombopag and hetrombopag may impose a higher administration burden. This could potentially lead to non-adherence and subsequent sub-therapeutic drug levels, which could compromise clinical effectiveness compared to the controlled trial setting. However, the substantial cost differential observed in our base case suggests that even if the “no fasting” benefit of avatrombopag resulted in slightly superior real-world effectiveness, it is unlikely to offset the economic advantage of hetrombopag. This is supported by our sensitivity analysis, where hetrombopag remained cost-effective even when relative efficacy parameters varied in a direction favorable to avatrombopag.

The exclusion of splenectomy from our model’s treatment pathway reflects specific clinical practices in China. Although international guidelines recommend splenectomy as a definitive option for refractory ITP (28, 29), its utilization in China has significantly declined. Cultural aversion to organ removal and concerns regarding surgical risks often render splenectomy a last-resort option rather than a standard second-line therapy. Consequently, TPO-RAs in China are not merely a “bridge” to surgery but often serve as lifelong maintenance therapy. This practice pattern underscores the importance of the lifetime horizon used in our model. In a system where patients may remain on pharmacological treatment for decades, the cumulative acquisition cost becomes the dominant driver of total healthcare expenditure, further solidifying the advantage of the lowest-cost efficacious agent, hetrombopag.

Several limitations of this study should be acknowledged. First, relative efficacy parameters were derived from an NMA (22) rather than head-to-head randomized controlled trials, introducing inherent uncertainty into the comparative estimates. Second, we assumed equivalent safety profiles across the three TPO-RAs, as recent systematic reviews indicated no statistically significant differences in overall adverse event rates (22). Third, the use of a standardized subsequent treatment regimen (rituximab + rhTPO) simplifies the heterogeneity of clinical practice across different regions in China, although this regimen aligns with current Chinese clinical guidelines (13). To address this uncertainty, we performed a scenario analysis varying the costs and efficacy of subsequent treatment by ±30% (Supplementary Table S5), which confirmed that the economic superiority of hetrombopag remained robust. Fourth, due to the lack of local data, utility values were derived from UK patients. Although cultural differences in health perception may introduce bias, sensitivity analyses indicated that variations in utility values were not key drivers of the model and did not alter the study’s conclusions. Fifth, the long-term extrapolation of response durability was based on eltrombopag data, assuming a class effect for the shape of the survival curve. Notably, our estimated mean duration of response for eltrombopag differs from prior estimates by Lee et al. (17), as our analysis incorporated the final results from the EXTEND study (16) rather than the interim data (30) used in previous models. This approach, while robust for eltrombopag, may not perfectly reflect the long-term trajectory of newer agents such as hetrombopag and avatrombopag. Finally, we did not explicitly quantify the utility decrement associated with the dietary restrictions of eltrombopag and hetrombopag, which may slightly favor these agents over avatrombopag in the utility analysis.

## 5 Conclusion

From the perspective of the Chinese healthcare system, hetrombopag represents the preferred strategy for the second-line treatment of adult chronic ITP, offering the optimal balance between cost and health outcomes. It dominates eltrombopag and provides a superior net monetary benefit compared with avatrombopag under current pricing conditions. These findings support the prioritization of hetrombopag in formulary decision-making and resource allocation, particularly in resource-constrained settings.

## Data Availability

The original contributions presented in the study are included in the article/Supplementary material, further inquiries can be directed to the corresponding authors.
