# Supplementary material for: Cost-effectiveness of hetrombopag, eltrombopag, and avatrombopag for chronic immune thrombocytopenia in China: a cost-utility analysis
Source: Front Public Health. 2026 Feb 19;14:1763592. doi: 10.3389/fpubh.2026.1763592 (PMC12960604; doi:10.3389/fpubh.2026.1763592)
Supplement: Supplementary file 1 [file Data_Sheet_1.docx]

Supplementary Material

**Table S1. Baseline Characteristics of the Model Cohort (1)**

| **Characteristic** | **Value(n=104)** |
| --- | --- |
| **Age, years** |  |
| Mean (SD) | 44.7(15.91) |
| Median (range) | 48(18–84) |
| **Gender, n (%)** |  |
| Female | 77(74.0) |
| Male | 27(26.0) |
| **Weight, kg** |  |
| Mean (SD) | 63.49 (11.98) |
| Median (range) | 62 (44-96) |
| **BMI, kg/m^2^** |  |
| Mean (SD) | 24.11 (3.58) |
| Median (range) | 23.8(17.2-34.9) |
| **Baseline stratification variables, n (%)** |  |
| Baseline ITP medication use | 53 (51.0) |
| Baseline platelet count ≤ 15 × 10^9/L | 54 (51.9) |
| Splenectomy | 18 (17.3) |
| Bleeding (WHO bleeding scale grade 1 - 4), n (%) | 68 (65.4) |
| Clinically significant bleeding (WHO bleeding scale grade 2 - 4), n (%) | 14 (13.5) |
| Median platelet count, × 10^9/L | 14.0 |
| ≥1 prior therapy, n (%) | 19 (18.3) |

BMI, body mass index; ITP, immune thrombocytopenia; SD, standard deviation; WHO, World Health Organization.

**Table S2. Dosing Schedules**

| **Medication** | **Mean Daily Dose** | **Frequency** | **Source** |
| --- | --- | --- | --- |
| **TPO-RAs** |  |  |  |
| Eltrombopag | 42.1 mg | Daily | (1) |
| Hetrombopag | 5.0 mg | Daily | Package Insert |
| Avatrombopag | 20.0 mg | Daily | Package Insert |
| **Subsequent Treatment** |  |  |  |
| Rituximab | 100 mg | Weekly (× 4) | (2) |
| rhTPO | 300 U·kg-1·d-1 | Daily (× 14) | (2) |

TPO-RAs, Thrombopoietin receptor agonists; rhTPO, recombinant human thrombopoietin.

**Table S3. Survival curve parameters**

| **Treatment** | **Shape Parameter** | **Scale Parameter** |
| --- | --- | --- |
| Eltrombopag | 3.44 | 0.70 |
| Rituximab + rhTPO | 2.68 | 1.48 |

The log-normal distribution was selected for the base-case analysis due to the lowest AIC and BIC values. rhTPO, recombinant human thrombopoietin.

**Table S4. Representative Background Mortality Rates (3)**

| **Age (Years)** | **Male (per 1,000)** | **Female (per 1,000)** |
| --- | --- | --- |
| 45-49 | 2.44 | 1.00 |
| 50-54 | 3.70 | 1.54 |
| 55-59 | 6.39 | 2.53 |
| 60-64 | 10.23 | 4.21 |
| 65-69 | 16.74 | 7.83 |
| 70-74 | 26.59 | 14.12 |
| 75-79 | 43.44 | 26.44 |

**Table S5. Scenario Analysis Results (iNMB in CNY)**

| **Comparisons** | **Subsequent Treatment Cost (-30%)** | **Subsequent Treatment Cost (+30%)** | **Subsequent Treatment Efficacy**  **(-30%)** | **Subsequent Treatment Efficacy**  **(+30%)** | **WTP Threshold**  **(1× GDP)** |
| --- | --- | --- | --- | --- | --- |
| Hetrombopag vs. Eltrombopag | 48,212 | 70,338 | 41,259 | 54,486 | 25,495 |
| Hetrombopag vs. Avatrombopag | 154,149 | 127,578 | 162,053 | 147,031 | 162,700 |
| Eltrombopag vs. Avatrombopag | 105,937 | 57,240 | 120,794 | 92,545 | 137,205 |

iNMB, incremental net monetary benefit; WTP, willingness-to-pay.

**Figure S1**


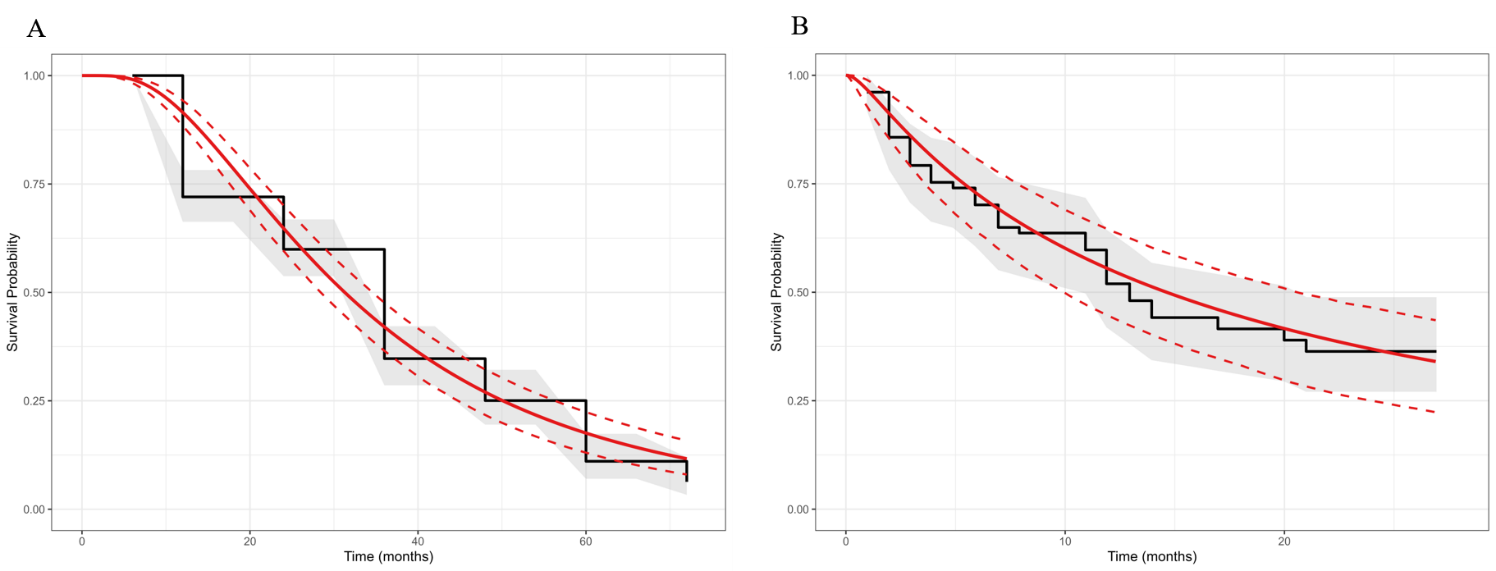


**Figure S1. Survival Curve Fitting for Duration of Response.** (A): Fitted curves of duration of response for eltrombopag. (B): Fitted curves for subsequent treatment duration. The solid black lines represent Kaplan-Meier estimates, and the red lines represent the parametric survival models used for extrapolation. The Log-normal distribution (red lines) provided the best fit.

**References**

1. Yang R, Li J, Jin J, Huang M, Yu Z, Xu X, et al. Multicentre, Randomised Phase iii Study of the Efficacy and Safety of Eltrombopag in Chinese Patients with Chronic Immune Thrombocytopenia. *Br J Haematol* (2017) 176(1):101-10. doi: 10.1111/bjh.14380.

2. Thrombosis and Hemostasis Group CSoH, Chinese Medical Association. Chinese Guideline on the Diagnosis and Management of Adult Primary Immune Thrombocytopenia (Version 2025). *Chin J Hematol* (2025) 46(12):1105-13. doi: 10.3760/cma.j.cn121090-20251031-00494.

3. National Health Commission of the People's Republic of China. *China Health Statistics Yearbook 2023*. Beijing: China Union Medical University Press (2023).
